# Cumulative exposure to high remnant-cholesterol concentrations increases the risk of cardiovascular disease in patients with hypertension: a prospective cohort study

**DOI:** 10.1186/s12933-023-01984-4

**Published:** 2023-09-21

**Authors:** Weiqiang Wu, Guanzhi Chen, Kuangyi Wu, Huancong Zheng, Yanjuan Chen, Xianxuan Wang, Zegui Huang, Zefeng Cai, Zhiwei Cai, Zhichao Chen, Yulong Lan, Shuohua Chen, Shouling Wu, Youren Chen

**Affiliations:** 1 Department of Cardiology, Second Affiliated Hospital of Shantou University Medical College, 69 Dongxia North RD, Shantou, 515000 China; 2 Shantou University Medical College, Shantou, China; 3 Cardiac Arrhythmia Center, Fuwai Hospital, National Center for Cardiovascular Diseases, Chinese Academy of Medical Sciences and Peking Union Medical College, Beijing, China; 4 Department of Endocrinology, Second Affiliated Hospital of Shantou University Medical College, Shantou, China; 5 Department of Cardiology, Kailuan General Hospital, 57 Xinhua East RD, Tangshan, 063000 China

**Keywords:** Remnant cholesterol, Cumulative exposure, Hypertension, Cardiovascular disease

## Abstract

**Background:**

The relationship of cumulative remnant-cholesterol (Cum-RC) concentration with the risk of cardiovascular disease (CVD) in patients with hypertension remains unclear.

**Methods:**

We studied data for 28,698 individuals for whom three consecutive total cholesterol, high-density lipoprotein-cholesterol (HDL-C), and triglyceride concentrations were available, and who did not have CVD (14,349 with hypertension and 14,349 without), that was collected between 2006 and 2010. Participants with hypertension were placed into four groups based on Cum-RC quartile: a Q1 group (< 26.40 mg/dl), a Q2 group (26.40–39.56 mg/dl), a Q3 group (39.57–54.65 mg/dl), and a Q4 group (≥ 54.66 mg/dl). Cox proportional hazards models were used to evaluate the relationship between Cum-RC and the risk of CVD.

**Results:**

Over a median 10.9 (interquartile range, 10.5–11.3) years, 1,444 participants with hypertension developed CVD. After adjustment for multiple potential confounding factors, and compared with the Q1 Cum-RC group of the participants with hypertension, the adjusted hazard ratios for CVD for the Q2–Q4 groups were 1.07(0.92,1.26), 1.08(0.91,1.28), and 1.26(1.03,1.54) (*P* = 0.0405); those for myocardial infarction were 1.51(1.00,2.31), 2.02(1.22,3.27), and 2.08(1.41,3.28) (*P* < 0.0001); and those for ischemic stroke were 1.02(0.84,1.24), 1.04(0.86,1.25), and 1.29(1.02,1.62), respectively (*P* = 0.0336). However, no significant relationship was found between Cum-RC and the risk of hemorrhage stroke. At the same Cum-RC, the risk of CVD was significantly higher in participants with hypertension than in those without.

**Conclusions:**

A consistently high remnant-cholesterol concentration increases the risk of CVD in individuals with hypertension. Therefore, the achievement of blood pressure and RC concentration targets should help reduce the risk of CVD in individuals with hypertension.

**Supplementary Information:**

The online version contains supplementary material available at 10.1186/s12933-023-01984-4.

## Background

Cardiovascular disease (CVD) is one of the leading causes of death worldwide [1]. In China, the incidences of CVD and CVD-related mortality continue to rise, and it accounts for two out of every five deaths [2]. Hypertension and abnormal lipid metabolism are major risk factors for CVD [1, 3–5]. Lewington et al. found that from blood pressure as low as 115/75 mmHg, the risk of developing CVD doubles for every increase in blood pressure of 20/10 mmHg [3]. Although LDL-C has been a particular focus during the past few decades, recent studies have shown that even after target LDL-C concentrations are achieved through statin therapy, the incidence of recurrence of cardiovascular events is high, which indicates that residual risk remains [6, 7]. Remnant cholesterol (RC) plays an important role in residual cardiovascular risk [8, 9] and can explain the risks of cardiovascular events and mortality that remain after target LDL-C concentrations are achieved [10]. Moreover, an increasing amount of evidence suggests that RC is more useful than LDL-C for the prediction of arterial sclerosis and the assessment of cardiovascular risk [10, 11].

RC increases the risk of CVD in people with either diabetes or pre-diabetes [12]. Previous studies have also shown that RC can accelerate the development of hypertension [13], and when hypertension and an abnormal circulating lipid profile coexist, the risk of CVD further increases [14]. However, previous studies of the combined effects of hypertension and abnormal lipid metabolism on CVD have principally analyzed the joint effects of LDL-C and blood pressure [14], and no studies have investigated the effect of RC in combination with hypertension on the risk of CVD. Therefore, to clarify the effect of this combination on the risk of CVD, we used cumulative remnant cholesterol (Cum-RC), an index that is potentially superior to a single measurement of RC, to analyze the effect of this lipid on the incidence of CVD in individuals with hypertension.

## Methods

### Study design and participants

**Fig. 1:**
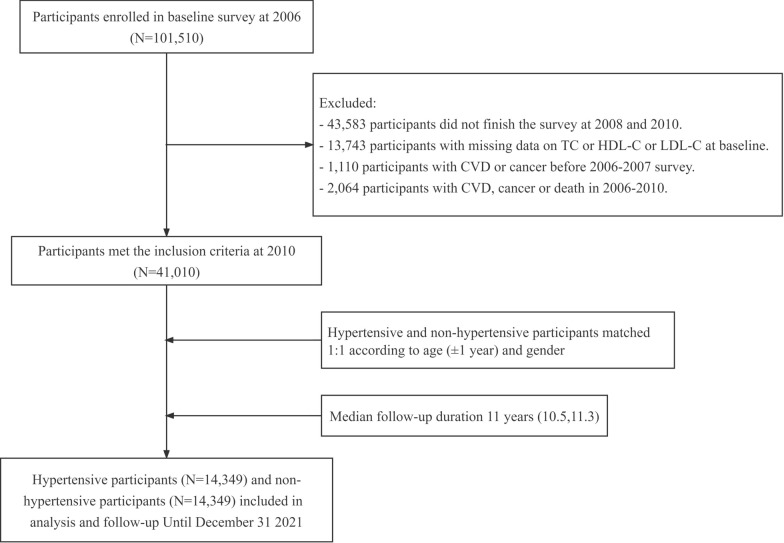
Flow chart for the inclusion of participants in the study

The Kailuan Study (registration number: ChiCTR-TNRC-11,001,489) is a prospective cohort study of the investigation of and interventions for CVD and related risk factors that is being conducted with community-based participants. A full description can be found in other articles published by our team [15, 16]. In 2006, investigators based at Kailuan General Hospital and its 10 affiliated hospitals conducted the first health examinations of 101,510 current or retired employees of the Kailuan Group, then follow-up examinations were conducted every 2 years thereafter, with the same measurements being made on each occasion. In the present study, to evaluate the cumulative effect of RC on CVD in participants with hypertension, we included only the participants who underwent health examinations in each of 2006, 2008, and 2010. The exclusion criteria were as follows: lack of participation in these three consecutive health examinations; missing TC, HDL-C, or LDL-C data during this period; and the development of CVD or cancer, or death, before 2010. Participants with hypertension who met the inclusion criteria and those without hypertension were matched in the baseline year of 2010 according to age (± 1 year) and sex at a ratio of 1:1. After the application of these criteria, a total of 14,349 participants with hypertension and an equal number of those without were identified, and they were followed until December 31, 2021. A flow diagram of the inclusion and exclusion process is provided in Fig. 1. The study was approved by the Ethics Committee of Kailuan General Hospital (approval number: 200,605) and was conducted according to the principles of the Declaration of Helsinki. Written informed consent was obtained from all the participants.

### Data collection

The details of the epidemiological survey and the anthropometric measurement made can be found in previous publications by our research group [15–17]. All the measurements were made in a quiet room at a temperature between 22℃ and 25℃. All the participants completed a questionnaire during each physical examination that collected information regarding their demographic characteristics (sex, age, and educational level), medical history (of hypertension, diabetes, and CVD; plus the use of antihypertensive drugs, glucose-lowering drugs, or lipid-lowering drugs), and lifestyle (smoking status, alcohol consumption habits, and exercise habits). When making anthropometric measurements, the participants were required to wear lightweight clothing and no shoes. Trained physicians measured their body mass and height according to standardized procedures, under standardized conditions, using calibrated instruments, to the nearest 0.1 kg and 0.1 cm, respectively. Body mass index (BMI) was calculated as body mass (kg) divided by height squared (m^2^). The blood pressure of the participants was measured in the right arm by an experienced physician using a calibrated mercury sphygmomanometer after a 15-minute rest. At least two blood pressure measurements were made and the mean value was recorded. However, if the difference between the two measurements was > 5 mmHg, another measurement was made, and the mean of the last two readings was recorded.

### Measurement of biochemical parameters

The participants were required to fast for more than 8 h, then venous blood samples were collected, and serum was separated by centrifugation. The serum concentrations of TC, LDL-C, HDL-C, TG, fasting glucose, and high-sensitivity C-reactive protein (hs-CRP) were measured using a Hitachi 7600 automatic biochemical analyzer (Tokyo, Japan), according to the kit manufacturers’ instructions, by professional laboratory technicians.

### Definition of remnant cholesterol

RC was calculated as TC − HDL-C − LDL-C [16]. The formula used for the calculation of the time-weighted mean Cum-RC was [(RC_2006_ + RC_2008_)/2×(Visit time_2008_ − Visit time_2006_)+(RC_2008_ + RC_2010_)/2×(Visit time_2010_ − Visit time_2008_)]/(Visit time_2010_ − Visit time_2006_); where Visit time_2006_, Visit time_2008_, and Visit Time_2010_ were the dates of follow-up during 2006, 2008, and 2010, respectively [18, 19]. The participants were placed into groups according to quartile of Cum-RC, as follows: Q1 (< 26.40 mg/dl), Q2 (26.40–39.56 mg/dl), Q3 (39.57–54.65 mg/dl), and Q4 (≥ 54.65 mg/dl).

### Other definitions

Hypertension was defined using a systolic blood pressure (SBP) ≥ 140 mmHg, and/or a diastolic blood pressure (DBP) ≥ 90 mmHg, and/or the use of an antihypertensive drug during the preceding 2 weeks [4]. According to the 2018 Revised Chinese Guidelines for the Prevention and Treatment of Hypertension, participants whose blood pressure was < 140/90 mmHg at all three physical examinations conducted between 2006 and 2010 were defined as having achieved blood pressure control, and those with any blood pressure reading ≥ 140/90 mmHg were defined as not having achieved control [20]. Diabetes was defined using an FPG ≥ 7.0 mmol/L, and/or a history of a clear diagnosis of diabetes, and/or the use of a hypoglycemic drug [21]. Users of lipid-lowering drugs were defined as those who were taking a statin, niacin, or a fibric acid derivative on a long-term basis [22]. A current smoker was defined as someone who had smoked on average ≥ 1 cigarette/day for > 1 year, and had smoked during the preceding year. A current alcohol consumer was defined as someone who had consumed on average ≥ 100 ml/day alcohol (alcohol content above 50%) for > 1 year, and had consumed alcohol during the preceding year. Educational level was defined as junior high school or below, or senior high school or above. Education was defined as senior high school or above. Participation in significant physical exercise was defined as the performance of exercise ≥ 3 times/week for ≥ 30 min on each occasion [23].

### Outcomes

The observation period started at the time of the physical examination in 2010, and the end point event was the development of CVD, including ischemic stroke, hemorrhagic stroke, and myocardial infarction. The diagnostic criteria used were those defined in the International Classification of Diseases 10th Revision (ICD-10), with I21 for myocardial infarction, I60 to I61 for hemorrhagic stroke, and I63 for ischemic stroke [24, 25]. Myocardial infarction was defined as elevated troponin T or troponin I with or without the following manifestations: ST-segment elevation on the electrocardiogram (ECG), or changes in myocardial ischemia on the ECG, or symptoms such as chest pain [26].Stroke is defined as a focal neurological deficit of sudden onset and vascular mechanism of > 24 h duration. The diagnosis of stroke was confirmed by a combination of brain computed tomography (CT) or magnetic resonance (MR) according to the World Health Organization (WHO) criteria and was classified as ischemic stroke and cerebral hemorrhage [27].For participants who experienced two or more events, the timing and type of the first event were recorded as the outcome, but if a participant experienced different types of cardiovascular event during the observation period, each event was recorded separately. For participants who did not experience any events, the observation period ended on December 31, 2021. Trained medical staff reviewed the diagnoses made upon admittance to hospitals affiliated with the Kailuan Group and designated medical insurance hospitals in Tangshan City every year, and recorded information regarding the endpoint events. The diagnoses were confirmed by physicians based on the hospital records.

### Statistical analysis

SAS 9.4 statistical software (Cary, NC, USA) was used for data analysis. Normally distributed continuous data are expressed as mean ± standard deviation, and groups were compared using one-way ANOVA. Skewed continuous data are expressed as median and interquartile range, and groups were compared using the Kruskal–Wallis test. Categorical data are expressed as numbers or percentages, and groups were compared using the chi-square test. The incidence of CVD in each Cum-RC group was calculated using the Kaplan–Meier method, and these were compared using the Log-rank test. Subsequently, we conducted a proportional hazards assumption, which suggested a p-value > 0.05. Cox proportional hazards regression models were used to evaluate the relationships of Cum-RC with the risks of CVD and its subtypes in patients with hypertension by calculating hazard ratios (HRs) and 95% confidence intervals (CIs). Model 1 was adjusted for age (continuous variable; years) and sex (categorical variable; male 1, female 0). Model 2 was further adjusted for smoking status (categorical variable, smoker or non-smoker), alcohol consumption (categorical variable, alcohol drinker or non-drinker), physical exercise habits (categorical variable, active or inactive), diabetes (categorical variable, present or absent), SBP (continuous variable, mmHg), HDL-C (continuous variable, mg/dl), LDL-C (continuous variable, mg/dl), hs-CRP (continuous variable, mg/L), BMI (continuous variable, kg/m^2^), eGFR (continuous variable, ml/min/1.73m^2^), family history of CVD (categorical variable, yes or no), the use of antihypertensive drugs (categorical variable, yes or no), the use of hypoglycemic drugs (categorical variable, yes or no), and the use of lipid-lowering drugs (categorical variable, yes or no). Model 3 was further adjusted for baseline RC (continuous variable, mg/dl). In addition, with non-hypertensive participants with Cum-RC < 26.40 mg/dl as the reference group, Cox proportional hazards regression models were used to evaluate the effect of the same Cum-RC on the risk of CVD in patients with hypertension, versus those without. These models were corrected for the variables listed above. Finally, stratified analyses were conducted based on age, sex, and whether the blood pressure of the participants was under control or not. To ensure the robustness of the results, sensitivity analyses were also conducted by excluding potential confounding factors (cardiovascular events within the first year of follow-up, the use of antihypertensive drugs, the use of lipid-lowering drugs). We then compared the risk of CVD in people with hypertension combined with diabetes and those with hypertension but no diabetes in the cox proportional hazards regression models. These models were corrected for the variables listed above. Finally we compared the ability of non-HDL-C, RC_2006_, RC_2010_, and Cum-RC to predict CVD risk in the China-PAR risk assessment model [28, 29]. *P* < 0.05 (two-tailed) was considered to represent statistical significance.

## Results

### Characteristics of the participants

A total of 14,349 participants with hypertension and 14,349 without were followed in the present study. The mean age of the participants was 56.05 ± 10.29 years, and men accounted for 82.0% of the participants in each group. The baseline clinical and biochemical characteristics of the participants with hypertension, grouped according to the quartile of Cum-RC, are shown in Table 1. The baseline clinical and biochemical characteristics of the non-hypertensive participants are shown in Additional file 1: Table S1, and a comparison between the baseline characteristics of the two groups is presented in Additional file 1: Table S2. Compared with the other three groups, the participants in the Q4 group, irrespective of the presence or absence of hypertension, were older; had higher BMI, SBP, and DBP; had higher fasting glucose, TG, RC, and hs-CRP concentrations; were more likely to have unhealthy habits (smoking, alcohol consumption, and physical inactivity); and had a higher prevalence of diabetes (*P* < 0.01) (Table 1).

**Table 1:** Baseline characteristics of participants with hypertension by cumulative RC quartiles

	Total	Q1 (< 26.40 mg/dl)	Q2 (26.40-39.56 mg/dl)	Q3 (39.57-54.65 mg/dl)	Q4 (≥ 54.66 mg/dl)	*P value
Participants	14,349	3587	3587	3588	3587	*/*
Age (years)	56.05 ± 10.29	55.59 ± 10.81	56.07 ± 9.38	56.11 ± 10.18	56.44 ± 10.74	0.02
Male, N (%)	11,771(82.0)	3041(84.8)	2974 (82.9)	2856(79.6)	2900(80.8)	< 0.01
BMI (kg/m^2^ )	26.06 ± 3.41	25.47 ± 3.34	26.17 ± 3.45	26.13 ± 3.46	26.45 ± 3.30	< 0.01
SBP (mmHg)	141.77 ± 19.08	142.09 ± 19.56	140.96 ± 19.42	141.85 ± 19.12	142.19 ± 8.16	< 0.01
DBP (mmHg)	89.66 ± 10.87	89.14 ± 10.93	89.63 ± 11.07	89.93 ± 10.45	89.95 ± 10.99	< 0.01
HDL-C (mg/dl)	57.44 ± 15.87	60.53 ± 15.43	56.71 ± 15.02	56.31 ± 15.72	56.21 ± 16.87	< 0.01
LDL-C (mg/dl)	101.20 ± 31.91	103.54 ± 27.54	104.99 ± 28.52	101.02 ± 32.71	95.25 ± 37.10	< 0.01
*TG (mg/dl)	53.90(37.73–80.85)	36.58(26.95–48.90)	53.90(40.43–73.54)	53.90(42.35–85.86)	77.00(51.98-120.89)	< 0.01
*RC_2006_(mg/dl)	45.43(25.03–71.61)	20.02(14.25–28.49)	38.50(26.56–52.75)	56.21(40.81–73.15)	84.70(62.37-112.42)	< 0.01
*RC_2008_(mg/dl)	32.34(19.63–53.90)	16.48(11.94–21.56)	29.26(22.33–36.96)	45.43(31.96–57.37)	67.76(46.20-87.01)	< 0.01
*RC_2010_(mg/dl)	29.65(16.56–54.29)	16.56(11.55–23.87)	26.95(16.56–41.20)	36.19(20.02–57.75)	61.98(37.35–93.17)	< 0.01
*Cum-RC(mg/dl)	39.63(26.49–55.49)	19.72(15.64–23.17)	32.92(29.79–36.04)	46.89(43.17–50.59)	68.70(61.03–80.43)	< 0.01
FPG (mmol/L)	5.92 ± 1.95	5.65 ± 1.48	5.87 ± 1.69	5.98 ± 1.79	6.18 ± 2.60	< 0.01
*hs-CRP (mg/L)	1.40(0.62–3.20)	1.40(0.70-3.00)	1.30(0.55-3.00)	1.40(0.60–3.26)	1.30(0.66–3.63)	< 0.01
eGFR(ml/min/1.73m^2^)	84.73 ± 18.76	79.39 ± 18.64	83.78 ± 18.58	87.06 ± 18.35	88.70 ± 18.10	< 0.01
Current smoker, N (%)	5434(37.9)	1127(31.4)	1389(38.7)	1443(40.2)	1475(41.1)	< 0.01
Alcohol drinker, N (%)	4999(34.8)	1037(28.9)	1233(34.4)	1331(37.1)	1398(39.0)	< 0.01
Physical activity, N (%)	2151(15.0)	509(14.2)	606(16.9)	564(15.7)	472(13.2)	< 0.01
Education, N (%)	2669(18.6)	591(16.5)	776(21.6)	681(19.0)	621(17.3)	< 0.01
Diabetes, N (%)	2335(16.3)	415(11.6)	535(14.9)	608(16.9)	777(21.7)	< 0.01
Antihypertensive drugs, N (%)	4372(30.5)	980(27.3)	1118(31.2)	1117(31.1)	1157(32.3)	< 0.01
Hypoglycemic drugs, N (%)	1164(8.11)	185(5.16)	266(7.42)	309(8.61)	404(11.3)	< 0.01
Lipid-lowering drugs, N (%)	195(1.36)	16(0.45)	39(1.09)	57(1.59)	83(2.31)	< 0.01

BMI body mass index, SBP systolic blood pressure, DBP diastolic blood pressure, HDL-C high-density lipoprotein cholesterol, LDL-C low-density lipoprotein cholesterol, FPG fasting plasma glucose, TG triglyceride, hs-CRP high-sensitivity C reactive protein,eGFR estimated glomerular filtration rate, RC remant cholesterol, Cum-RC cumulative remant cholesterol

*P value, comparison of baseline characteristics between diferent Cum-RC groups

*TG, hs-CRP, RC_2006_, RC_2008_, RC_2010_ and Cum-RC were expressed by median (IQR)

### Relationships of Cum-RC with the incidences of CVD and its subtypes in the participants with hypertension

**Fig. 2:**
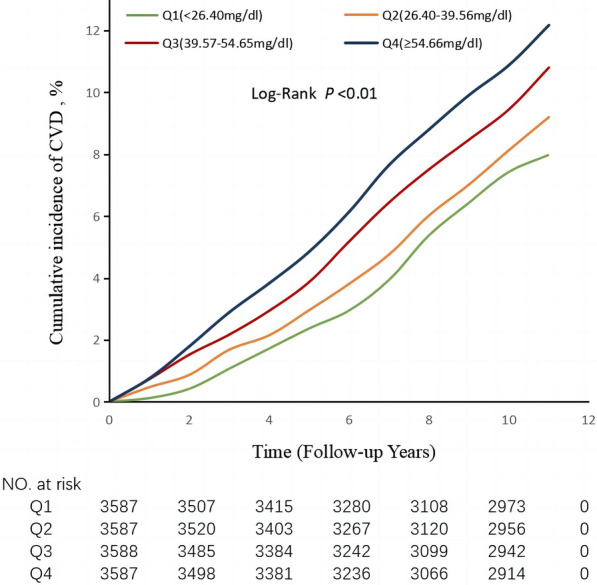
Kaplan–Meier analysis of cumulative RC and incidence rate of CVD in participants with hypertension

During a median follow-up period of 10.9 years (interquartile range, 10.4 to 11.3 years), 1,444 (10.06%) participants with hypertension experienced CVD events. During the observation period, there were 283 cases of myocardial infarction, 1,055 cases of ischemic stroke, and 148 cases of hemorrhagic stroke among the participants with hypertension. The cumulative incidences of CVD in the various groups of participants with hypertension significantly differed, according to the log-rank test (*P* < 0.01; Fig. 2). The incidence of CVD increased across the Q1–Q4 groups of the participants with hypertension: it was 8.77, 9.65, 9.86, and 11.87 per 1,000 person-years, respectively (Table 2). After adjustment for potentially confounding factors in Model 3, compared with the Q1 Cum-RC group of participants with hypertension, the adjusted HRs (95% CIs) for CVD for the Q2–Q4 groups were 1.07(0.92,1.26), 1.08(0.91,1.28), and 1.26(1.03,1.54), respectively (*P* = 0.0405). The adjusted HRs (95% CIs) for myocardial infarction were 1.51(1.00, 2.31), 2.02(1.22, 3.27), and 2.08(1.41, 3.28), respectively (*P* < 0.0001); those for ischemic stroke were 1.02(0.84, 1.24), 1.04(0.86, 1.25), and 1.29(1.02, 1.62) (*P* = 0.0336); and those for hemorrhagic stroke were 1.06(0.68, 1.71), 0.86(0.50, 1.46), and 0.97(0.51, 1.84), respectively(*P* = 0.7247) (Table 2).

**Table 2:** Association of cumulative RC with CVD and subtypes in participants with hypertension

Quartiles of Cum-RC	Case/Total	Incidence density, per 1000 person-years	Model 1	Model 2	Model 3
Cardiovascular disease					
Q1	317/3587	8.77	1.00	1.00	1.00
Q2	348/3587	9.65	1.15(0.99,1.34)	1.08(0.93,1.26)	1.07(0.92,1.26)
Q3	354/3588	9.86	1.19(1.02,1.39)	1.09(0.94,1.28)	1.08(0.91,1.28)
Q4	425/3587	11.87	1.43(1.24,1.65)	1.29(1.10,1.50)	1.26(1.03,1.54)
* P*-trend			< 0.0001	0.0015	0.0405
Myocardial infarction					
Q1	38/3587	1.02	1.00	1.00	1.00
Q2	61/3587	1.64	1.71(1.14,2.56)	1.57(1.05,2.37)	1.51(1.00,2.31)
Q3	88/3588	2.38	2.52(1.72,3.70)	2.30(1.56,3.39)	2.02(1.22,3.27)
Q4	96/3587	2.58	2.74(1.88,3.99)	2.48(1.68,3.66)	2.08(1.41,3.28)
* P*-trend			< 0.0001	< 0.0001	< 0.0001
Ischemic Stroke					
Q1	248/3587	6.79	1.00	1.00	1.00
Q2	253/3587	6.93	1.07(0.90,1.28)	1.00(0.84,1.20)	1.02(0.84,1.24)
Q3	258/3588	7.04	1.08(0.91,1.29)	1.02(0.86,1.22)	1.04(0.86,1.25)
Q4	296/3587	8.61	1.33(1.13,1.58)	1.23(1.04,1.47)	1.29(1.02,1.62)
* P*-trend			0.0012	0.0249	0.0336
Hemorrhagic stroke					
Q1	40/3587	1.02	1.00	1.00	1.00
Q2	40/3587	1.02	1.03(0.66,1.59)	1.04(0.67,1.62)	1.06(0.68,1.71)
Q3	32/3588	0.81	0.82(0.52,1.31)	0.79(0.49,1.28)	0.86(0.50,1.46)
Q4	36/3587	0.91	0.92(0.59,1.45)	0.83(0.52,1.34)	0.97(0.51,1.84)
* P*-trend			0.5254	0.2886	0.7247

Model 1: adjusted for age and sex

Model 2: included variables in model 1 and further SBP, LDL-C, HDL-C, BMI, hs-CRP, eGFR, family history of CVD, smoking status, alcohol consumption, physical exercise habits, diabetes, the use of antihypertensive drugs, the use of glucose-lowering drugs, and the use of lipid-lowering drugs

Model 3: included variables in model 2 and further the RC at baseline

### Comparison of the effects of Cum-RC on the risk of CVD in participants with and without hypertension

During a median follow-up period of 10.9(IQR 10.5,11.3) years, 867 (6.04%) participants without hypertension developed CVD. When they were grouped according to quartile of Cum-RC, their incidences of CVD were 5.32, 5.50, 5.42, and 7.25 per 1,000 person-years for the G1 (< 26.40 mg/dl), G2 (26.40–39.56 mg/dl), G3 (39.57–54.65 mg/dl), and G4 (≥ 54.66 mg/dl) groups, respectively (Table 3). Compared with the < 26.40 mg/dl group, the adjusted HRs (95% CIs) for the participants without hypertension in the 26.40–39.56 mg/dl, 39.57–54.65 mg/dl, and ≥ 54.66 mg/dl groups were 1.00 (0.82, 1.22), 0.95 (0.78, 1.17), and 1.27(1.03, 1.57), respectively (*P* < 0.0001) (Table 3). In addition, the adjusted HRs (95% CIs) for the participants with hypertension were 1.28 (1.06, 1.53), 1.38 (1.15, 1.66), 1.42 (1.17, 1.71), and 1.63 (1.32, 2.00), respectively (*P* < 0.0001) (Table 3).

**Table 3:** Association of cumulative RC with CVD in hypertensive participants and non-hypertensive participants

Cum-RC	Case/Total	Incidence density, per 1000 person-years	Model 1	Model 2	Model 3
G4*(≥ 54.66 mg/dl)	257/3433	7.25	1.45(1.21,1.75)	1.30(1.08,1.57)	1.27(1.03,1.57)
G3*(39.57-54.65 mg/dl)	209/3724	5.42	1.05(0.87,1.28)	0.97(0.79,1.17)	0.95(0.78,1.17)
G2*(26.40-39.56 mg/dl)	203/3583	5.50	1.08(0.89,1.31)	1.01(0.83,1.23)	1.00(0.82,1.22)
G1*(< 26.40 mg/dl)	198/3609	5.32	1.00 (Ref.)	1.00 (Ref.)	1.00 (Ref.)
G1(< 26.40 mg/dl)	317/3587	8.77	1.65(1.38,1.97)	1.28(1.06,1.53)	1.28(1.06,1.53)
G2(26.40-39.56 mg/dl)	348/3587	9.65	1.91(1.60,2.27)	1.39(1.16,1.67)	1.38(1.15,1.66)
G3(39.57-54.65 mg/dl)	354/3588	9.86	2.00(1.67,2.38)	1.44(1.20,1.72)	1.42(1.17,1.71)
G4(≥ 54.66 mg/dl)	425/3587	11.87	2.35(1.99,2.78)	1.67(1.40,1.99)	1.63(1.32,2.00)
*P*-trend			< 0.0001	< 0.0001	< 0.0001

G1*-G4*were participants without hypertension;G1-G4 were participants with hypertension

Model 1: adjusted for age and sex

Model 2: included variables in model 1 and further SBP, LDL-C, HDL-C, BMI, hs-CRP, eGFR, family history of CVD, smoking status, alcohol consumption, physical exercise habits, diabetes, the use of antihypertensive drugs, the use of glucose-lowering drugs, and the use of lipid-lowering drugs

Model 3: included variables in model 2 and further the RC at baseline

### Results of the stratified and sensitivity analyses

**Fig. 3:**
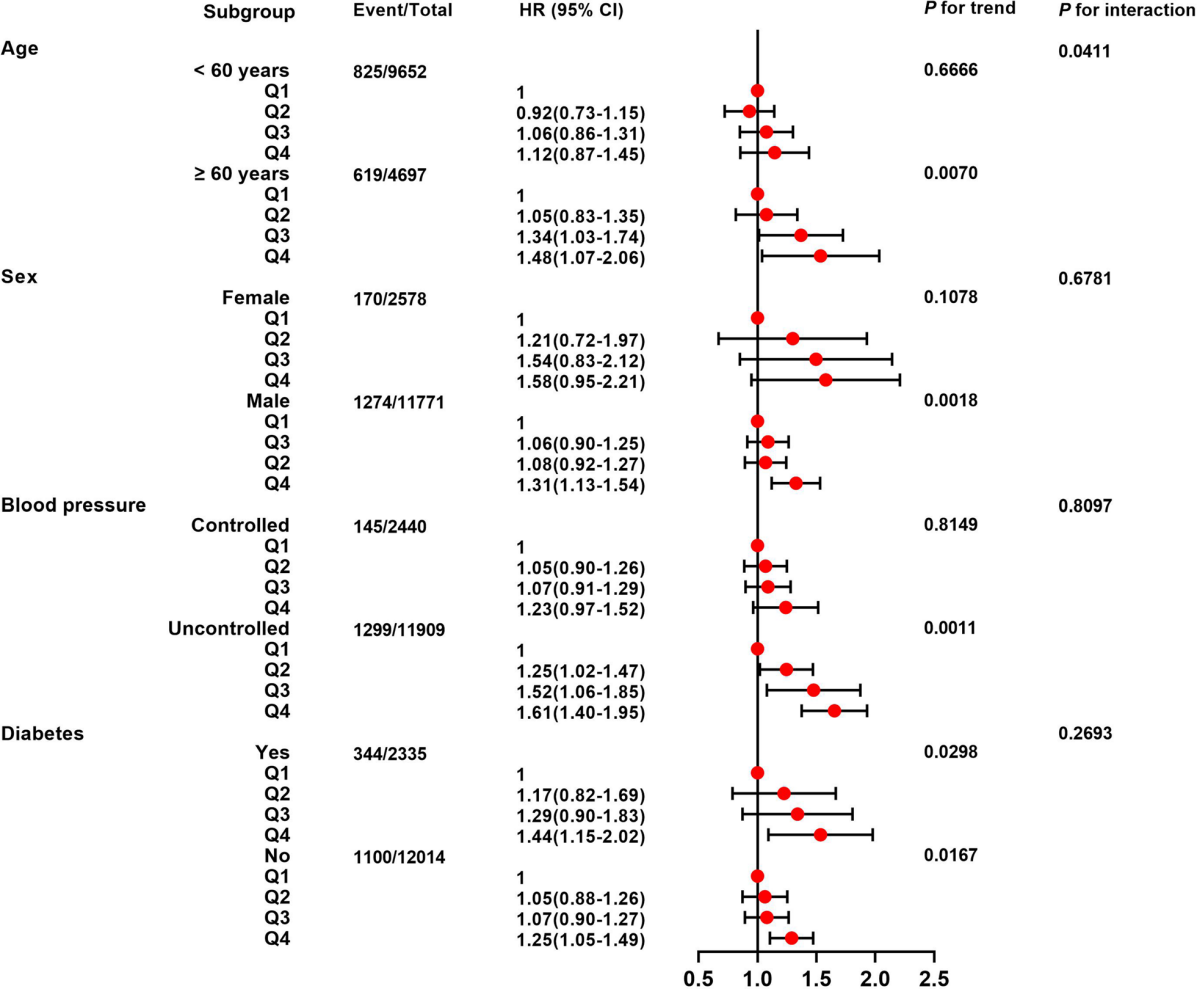
Stratified analysis of the relationship between cumulative RC and CVD in participants with hypertension The data were adjusted for age, sex, SBP, LDL-C, HDL-C, BMI, hs-CRP, eGFR, family history of CVD, smoking status, alcohol consumption habits, physical exercise habits, diabetes, the use of antihypertensive drugs, the use of glucose-lowering drugs, the use of lipid-lowering drugs, and the RC at baseline

The stratified analysis revealed that there was an interaction between Cum-RC and age, but none with sex or blood pressure control status. In participants aged ≥ 60 years, male, and those with poorly controlled blood pressure, high Cum-RC was associated with a higher risk of CVD; but there were no statistically significant associations in participants aged < 60 years, female, or in those with well-controlled blood pressure (Fig. 3).

To reduce the effects of potential confounders on the findings, the Cox regression analysis was repeated after the exclusion of participants who experienced CVD events within the first year of follow-up (161 individuals), those who were taking antihypertensive medication (4,372 individuals), those who were taking lipid-lowering medication (195 individuals). The results showed a significant association between high Cum-RC and a higher risk of CVD (Additional file 1: Table S3). At the same level of Cum-RC, the risk of CVD was significantly higher in those with hypertension combined with diabetes compared with those with hypertension but no diabetes (Additional file 1: Table S4).

### Comparison of the abilities of non-HDL-C, RC_2006_, RC_2010_, and Cum-RC to predict the risk of CVD

In the China-PAR risk assessment model, The C-index values for non-HDL-C, RC in 2006, RC in 2010, and Cum-RC were 0.6546, 0.6573, 0.6554, and 0.7028, respectively; the NRI values were 8.03%, 9.42%, 8.85% and 11.83%, respectively; and the IDI values were 0.017%, 0.036%, 0.030% and 0.044%; respectively (Additional file 1: Table S5). Cum-RC demonstrated significantly higher predictive ability for the risk of CVD than non-HDL-C, RC in 2006 and RC in 2010.

## Discussion

In the present study, we found a significant association between high Cum-RC exposure and a higher risk of CVD in individuals with hypertension, independent of conventional risk factors and the baseline concentration. In addition, the CVD risk attributable to the same Cum-RC exposure level was significantly higher in individuals with hypertension than in those without.

This association between RC and the risk of CVD has also been shown in previous studies [9, 30–33]. Fu et al. found that patients with type 2 diabetes are at a higher risk of major adverse cardiovascular events (MACEs) when the RC concentration is ≥ 31 mg/dl, regardless of the LDL-C concentration [33]. A cohort study of 1,956,452 patients with type 2 diabetes conducted in Korea showed that those in the highest quartile of RC were at a 28% higher risk of myocardial infarction and a 22% higher risk of ischemic stroke versus those in the lowest quartile [34]. In addition, a multi-cohort study showed higher risk of peripheral artery disease (PAD), myocardial infarction, and ischemic stroke in individuals with RC > 58 mg/dL, compared to those with RC < 19 mg/dL [35]. However, previous studies of the relationship between RC and CVD have been conducted in the general population or in patients with diabetes, and no studies have explored the effect of cumulative RC concentration on the risk of CVD in people with hypertension. We found a 26% higher risk of CVD in participants with hypertension in the fourth quartile of Cum-RC, versus those in the first quartile of Cum-RC, along with a 1.08-fold higher risk of myocardial infarction and a 29% higher risk of ischemic stroke, but we did not identify a relationship between Cum-RC and the risk of hemorrhagic stroke. Notably, hypertension is an independent risk factor for hemorrhagic stroke [36], but the results of a previous meta-analysis suggested that hyperlipidemia may help prevent hemorrhage, with a 7% reduction in the risk of hemorrhagic stroke for every 1 mmol/L increase in TG [37]. Therefore, the combination of these two opposing factors may abrogate the relationship between high Cum-RC exposure and hemorrhagic stroke in individuals with hypertension. In contrast to previous studies, in which only a single measurement of RC was made, we used the Cum-RC, and also a follow-up period of 11 years, which should render the results more reliable and relevant to real-world conditions than those of previous studies.

Another important finding of the present study was that at the same Cum-RC, the risk of CVD was significantly higher in participants with hypertension than in those without. Participants with hypertension and a Cum-RC ≥ 54.66 mg/dl were at a 63% higher risk of CVD than those without hypertension and a Cum-RC < 26.40 mg/dl, while participants without hypertension and a Cum-RC ≥ 54.66 mg/dl were at a 28% higher risk of CVD. At the same level of Cum-RC, the risk of CVD was significantly higher in those with hypertension combined with diabetes compared with those with hypertension but no diabetes. Previous TNT (Treating to New Targets) trial have shown that high TRL-C, along with high RC concentration, is associated with a higher risk of CVD, and that taking statins can significantly reduce the risk of coronary heart disease in patients with high RC-related TRL-C [38]. This suggests that the lipid profiles of patients with hypertension and diabetes should be assessed, and that all of their blood pressure, glycemic and lipid concentrations should be controlled to reduce the risk of CVD.

The stratified analysis showed that a high Cum-RC increases the risk of CVD in individuals with hypertension who are aged ≥ 60 years, are male, diabetes and have poor blood pressure control. In subsequent sensitivity analyses, we eliminated factors that might have affected this finding, such as the development of CVD within the first year of follow-up, the use of antihypertensive drugs, the use of lipid-lowering drugs; and the results still showed that high Cum-RC increased the risk of CVD in the participants with hypertension. These results suggests that the administration of antihypertensive drugs or lipid-lowering drugs and good blood pressure control may attenuate the effect of high Cum-RC to increase the risk of CVD in individuals with hypertension. Given the current low prevalence of good blood pressure control in patients with hypertension, increases in the use of medication and compliance in patients with hypertension and the control of risk factors, such as dyslipidemia and hyperglycemia, should be the main measures used to reduce the risk of CVD in such patients.

The mechanism linking RC and CVD may be related to the higher levels of lipoprotein-cholesterol present in RC than in LDL-C [39], which may activate vascular endothelial cells and induce endothelial dysfunction [40]. Furthermore, when both LDL-C and RC enter the arterial wall [41], RC is more readily absorbed by macrophages without oxidation than unmodified LDL-C, thereby increasing cholesterol accumulation [42]; and high RC concentrations are highly positively associated with low-grade inflammation [43]. However, the effect of hypertension on CVD is the result of a combination of genetic and environmental factors. Hypertension is a complex, multifactorial disease state that can involve a combination of pathophysiologic processes, including salt sensitivity, altered arterial compliance, altered vascular reactivity, and altered activity of the renin-angiotensin and adrenergic systems [44, 45]. A combination of abnormal RC and hypertension may promote atherosclerosis, impaired arterial compliance, vascular endothelial dysfunction, chronic inflammatory responses, and vascular remodeling, which are involved in the pathogenesis of CVD [40, 42, 43, 46].

The present study had the following strengths. First, it was the first prospective cohort study to show a longitudinal association between high Cum-RC exposure and the risk of CVD in individuals with hypertension. Second, the data were collected during a prospective cohort study with a sample size of 101,510 and an 11-year follow-up period, which renders them reliable. However, this study also had some limitations. First, because the Kailuan study participants are predominantly male, and active or retired Kailuan Group workers, there was some selection bias. Second, because it was a cohort study, we were unable to meticulously explore the pathophysiological mechanisms underlying the effect of RC abnormalities on CVD in the participants with hypertension or to accurately differentiate primary and secondary hypertension in each individual. Third, there may have been confounding factors that were not identified, such as the genetic susceptibility of the participants with hypertension. Fourthly, RC showed a gradual decrease with time. However, the use of time-weighted Cum-RC may solve the problem of gradual decrease in RC. Therefore, we corrected for confounding factors in the Cox regression model as far as possible, and also performed a sensitivity analysis. In addition, the results should be relatively reliable, owing to the large sample size and the long follow-up period used.

## Conclusions

In the present study, we found that high Cum-RC increases the risk of CVD in patients with hypertension, and that the risk of CVD associated with the same Cum-RC exposure is significantly higher in people with hypertension than in those without. These results suggest that repeated monitoring and maintenance of appropriate RC and blood pressure levels and the simultaneous control of blood pressure and RC may help to reduce the risk of CVD.

### Supplementary Information

**Additional file 1:** **Table S1. **Baseline characteristics of participants without hypertension by cumulative RC quartiles. **Table S2. **Baseline characteristics by non-hypertensive and hypertensive populations. **Table S3. **Sensitivity analysis for association of Cum-RC and CVD in participants with hypertension. **Table S4. **Association of cumulative RC with CVD in hypertensive participants with diabetic and non-diabetic. **Table S5. **Comparison of predictive ability for CVD between non-HDL-C, RC_2006_, RC_2010_ and Cum-RC.

## Data Availability

The datasets used and analyzed during the current study are available from the corresponding author on reasonable request.

## Abbreviations

RC: Remnant-cholesterol
Cum-RC: Cumulative remnant-cholesterol
CVD: Cardiovascular disease
TG: Triglyceride
FPG: Fasting plasma glucose
BMI: Body mass index
BP: Blood pressure
SBP: Systolic blood pressure
DBP: Diastolic blood pressure
TC: Total cholesterol
HDL-C: High-density lipoprotein-cholesterol
hs-CRP: High-sensitivity C-reactive protein
LDL-C: Low-density lipoprotein-cholesterol
eGFR: Estimated glomerular filtration rate
HR: Hazard ratio
CI: Confidence interval
