# Supplementary material for: Cumulative exposure to high remnant-cholesterol concentrations increases the risk of cardiovascular disease in patients with hypertension: a prospective cohort study
Source: Cardiovasc Diabetol. 2023 Sep 21;22:258. doi: 10.1186/s12933-023-01984-4 (PMC10515262; doi:10.1186/s12933-023-01984-4)
Supplement: Supplementary file 1 — Additional file 1: Table S1. Baseline characteristics of participants without hypertension by cumulative RC quartiles. Table S2. Baseline characteristics by non-hypertensive and hypertensive populations. Table S3. Sensitivity analysis for association of Cum-RC and CVD in participants with hypertension. Table S4. Association of cumulative RC with CVD in hypertensive participants with diabetic and non-diabetic. Table S5. Comparison of predictive ability for CVD between non-HDL-C, RC2006, RC2010 and Cum-RC. [file 12933_2023_1984_MOESM1_ESM.docx]

**Table S1** Baseline characteristics of participants without hypertension by cumulative RC quartiles

|  | **Total** | **Q1**  (<26.30mg/dl) | **Q2**  (26.31-39.50mg/dl) | **Q3**  (39.51-53.92mg/dl) | **Q4**  (≥53.92mg/dl) | *P **value** |
| --- | --- | --- | --- | --- | --- | --- |
| Participants | 14349 | 3587 | 3587 | 3588 | 3587 | */* |
| Age (years) | 56.05±10.29 | 55.82±9.34 | 55.94±10.71 | 56.21±9.88 | 56.22±11.14 | 0.42 |
| Male, N (%) | 11771(82.0) | 3035(84.6) | 2944(82.1) | 2932(81.7) | 2860(79.7) | <0.01 |
| BMI (kg/m^2^) | 24.75±3.15 | 24.17±3.05 | 24.79±3.13 | 24.81±3.16 | 25.25±3.16 | <0.01 |
| SBP (mmHg) | 126.81±16.12 | 126.04±16.47 | 126.54±16.16 | 126.90±15.94 | 127.74±15.86 | <0.01 |
| DBP (mmHg) | 81.98±9.43 | 80.73±9.22 | 81.85±9.37 | 82.49±9.55 | 82.86±9.44 | <0.01 |
| HDL-C (mg/dl) | 59.81±16.89 | 61.72±15.96 | 59.74±16.87 | 59.08±16.91 | 58.70±17.61 | <0.01 |
| LDL-C (mg/dl) | 101.06±30.79 | 100.47±27.37 | 101.88±28.30 | 101.55±31.27 | 100.35±35.53 | <0.01 |
| *****TG (mg/dl) | 48.13(33.88-72.00) | 35.04(25.80-46.97) | 47.36(35.04-65.45) | 52.36(36.58-76.62) | 66.61(45.43-107.80) | <0.01 |
| *****RC_2006_(mg/dl) | 46.20(25.80-68.91) | 19.63(13.86-28.88) | 40.81(27.34-56.60) | 55.06(40.42-70.84) | 76.62(58.52-100.49) | <0.01 |
| *****RC_2008_(mg/dl) | 33.50(19.25-56.98) | 15.02(10.78-20.79) | 28.49(21.56-37.73) | 48.13(33.88-58.91) | 68.15(50.82-84.32) | <0.01 |
| *****RC_2010_(mg/dl) | 26.18(14.63-49.67) | 16.17(10.78-23.87) | 23.10(13.86-38.50) | 29.26(15.79-50.82) | 55.83(32.73-84.32) | <0.01 |
| *****Cum-RC(mg/dl) | 39.51(26.30-53.92) | 18.87(14.84-22.77) | 32.98(29.75-36.28) | 46.26(42.79-49.62) | 65.48(58.91-76.22) | <0.01 |
| FPG (mmol/L) | 5.65±1.65 | 5.47±1.25 | 5.64±1.66 | 5.70±1.93 | 5.79±1.67 | <0.01 |
| *****hs-CRP (mg/L) | 1.18(0.60-2.59) | 1.19(0.60-2.60) | 1.10(0.52-2.40) | 1.10(0.58-2.60) | 1.22(0.60-2.70) | <0.01 |
| eGFR(ml/min/1.73m^2^) | 90.07±17.76 | 84.64±20.12 | 90.25±17.56 | 92.32±15.88 | 93.08±15.91 | <0.01 |
| Current smoker, N (%) | 5956(41.51) | 1211(34.04) | 1496(41.71) | 1609(44.80) | 1640(45.7) | <0.01 |
| Alcohol drinker, N (%) | 5401(37.64) | 1117(31.14) | 1415(39.45) | 1432(39.92) | 1437(40.1) | <0.01 |
| Physical activity, N (%) | 2332(16.25) | 570(15.89) | 594(16.56) | 621(17.31) | 547(15.25) | 0.06 |
| Education, N (%) | 3214(22.40) | 790(22.24) | 890(24.81) | 789(21.99) | 745(20.77) | <0.01 |
| Diabetes, N (%) | 1470(10.24) | 274(7.64) | 354(9.87) | 390(10.87) | 452(12.60) | <0.01 |
| Glucose-lowering drugs, N (%) | 793(5.53) | 134(3.74) | 182(5.07) | 221(6.16) | 256(7.14) | <0.01 |
| Lipid-lowering drugs, N (%) | 80(0.56) | 9(0.25) | 14(0.39) | 19(0.53) | 38(1.06) | <0.01 |

Note: *****P value, comparison of baseline characteristics between different Cum-RC groups.

BMI body mass index, SBP systolic blood pressure, DBP diastolic blood pressure, HDL-C high-density lipoprotein cholesterol, LDL-C low-density lipoprotein cholesterol, FPG fasting plasma glucose, TG triglyceride, hs-CRP high-sensitivity C reactive protein, eGFR estimated glomerular filtration rate, RC remnant cholesterol, Cum-RC cumulative remnant cholesterol.

*TG, hs-CRP, RC_2006_, RC_2008_, RC_2010_ and Cum-RC were expressed by median (IQR).

**Table S2** Baseline characteristics by non-hypertensive and hypertensive populations

|  | **Total** | **Non-hypertensive participants** | **Hypertensive participants** | ***P value** |
| --- | --- | --- | --- | --- |
| Participants | 28698 | 14349 | 14349 | / |
| Age (years) | 56.05±10.29 | 56.05±10.29 | 56.05±10.29 | 0.97 |
| Male, N (%) | 23542(82.03) | 11771(82.03) | 11771(82.03) | 1.00 |
| BMI (kg/m^2^ ) | 25.41±3.35 | 24.75±3.15 | 26.06±3.41 | <0.01 |
| SBP (mmHg) | 134.29±19.18 | 126.81±16.12 | 141.77±19.08 | <0.01 |
| DBP (mmHg) | 85.82±10.87 | 81.98±9.43 | 89.66±10.87 | <0.01 |
| HDL-C (mg/dl) | 58.63±16.43 | 59.81±16.89 | 57.44±15.87 | <0.01 |
| LDL-C (mg/dl) | 101.13±31.35 | 101.06±30.79 | 101.20±31.91 | 0.45 |
| *****TG (mg/dl) | 50.82(35.42-76.62) | 48.13(33.88-72.00) | 53.90(37.73-80.85) | <0.01 |
| *****RC_2006_(mg/dl) | 45.82(25.41-70.07) | 46.20(25.80-68.91) | 45.43(25.03-71.61) | 0.15 |
| *****RC_2008_(mg/dl) | 32.73(19.25-55.44) | 33.50(19.25-56.98) | 32.34(19.63-53.90) | 0.31 |
| *****RC_2010_(mg/dl) | 28.11(15.40-51.98) | 26.18(14.63-49.67) | 29.65(16.56-54.29) | <0.01 |
| *****Cum-RC(mg/dl) | 39.56(26.39-54.66) | 39.51(26.30-53.92) | 39.63(26.49-55.49) | <0.01 |
| FPG (mmol/L) | 5.78±1.81 | 5.65±1.65 | 5.92±1.95 | <0.01 |
| *****hs-CRP (mg/L) | 1.30(0.60-2.90) | 1.18(0.60-2.59) | 1.40(0.62-3.20) | <0.01 |
| eGFR(ml/min/1.73m^2^) | 87.40±18.46 | 90.07±17.76 | 84.73±18.76 | <0.01 |
| Current smoker, N (%) | 11390(39.69) | 5956(41.51) | 5434(37.87) | <0.01 |
| Alcohol drinker, N (%) | 10400(36.24) | 5401(37.64) | 4999(34.84) | <0.01 |
| Physical activity, N (%) | 4483(15.62) | 2332(16.25) | 2151(14.99) | <0.01 |
| Education, N (%) | 5883(20.50) | 3214(22.40) | 2669(18.60) | <0.01 |
| Diabetes, N (%) | 3805(13.26) | 1470(10.24) | 2335(16.27) | <0.01 |
| Antihypertensive drugs, N (%) | 4372(15.23) | 0(0.00) | 4372(30.47) | <0.01 |
| Glucose-lowering drugs, N (%) | 1957(6.82) | 793(5.53) | 1164(8.11) | <0.01 |
| Lipid-lowering drugs, N (%) | 275(0.96) | 80(0.56) | 195(1.36) | <0.01 |

Note: *****P vaulue, comparison of baseline characteristics between non-hypertensive and hypertensive populations.

BMI body mass index, SBP systolic blood pressure, DBP diastolic blood pressure, HDL-C high-density lipoprotein cholesterol, LDL-C low-density lipoprotein cholesterol, FPG fasting plasma glucose, TG triglyceride, hs-CRP high-sensitivity C reactive protein, eGFR estimated glomerular filtration rate, RC remant cholesterol，Cum-RC cumulative remant cholesterol.

*****TG, hs-CRP, RC_2006_, RC_2008_, RC_2010_ and Cum-RC were expressed by median (IQR).

**Table S3** Sensitivity analysis for association of Cum-RC and CVD in participants with hypertension

| **Cum-RC** | Analysis 1 | Analysis 2 | Analysis 3 |
| --- | --- | --- | --- |
| Q1 | 1.00 | 1.00 | 1.00 |
| Q2 | 1.07(0.91,1.26) | 1.12(0.93,1.35) | 1.09(0.93,1.27) |
| Q3 | 1.08(0.91,1.29) | 1.20(1.00,1.44) | 1.13(0.96,1.32) |
| Q4 | 1.30(1.06,1.61) | 1.35(1.12,1.63) | 1.33(1.14,1.55) |
| P-trend | 0.0216 | 0.0013 | 0.0003 |

Analysis 1：Excluded participants who developed CVD within 1 year of follow-up (n=161).

Analysis 2：Excluded participants with antihypertensive drugs at baseline (n=4372).

Analysis 3：Excluded participants with lipid-lowering drugs at baseline (n=195).

Analysis 1：Model adjusted for age and sex, SBP, LDL-C, HDL-C, BMI, hs-CRP, eGFR, family history of CVD, smoking status, alcohol consumption, physical exercise habits, diabetes, the use of antihypertensive drugs, the use of glucose-lowering drugs, the use of lipid-lowering drugs, and the RC at baseline.

Analysis 2：Model adjusted for age and sex, SBP, LDL-C, HDL-C, BMI, hs-CRP, eGFR, family history of CVD, smoking status, alcohol consumption, physical exercise habits, diabetes, the use of glucose-lowering drugs, the use of lipid-lowering drugs, and the RC at baseline.

Analysis 3：Model adjusted for age and sex, SBP, LDL-C, HDL-C, BMI, hs-CRP, eGFR, family history of CVD, smoking status, alcohol consumption, physical exercise habits, diabetes, the use of antihypertensive drugs, the use of glucose-lowering drugs, and the RC at baseline.

**Table S4** Association of cumulative RC with CVD in hypertensive participants with diabetic and non-diabetic

| **Cum-RC** | **Case/Total** | **Incidence density, per 1000 person-years** | **Model 1** | **Model 2** | **Model 3** |
| --- | --- | --- | --- | --- | --- |
| G4*(≥54.66mg/dl) | 309/2936 | 10.43 | 1.32(1.12,1.56) | 1.24(1.05,1.47) | 1.21(0.98,1.50) |
| G3*(39.57-54.65mg/dl) | 253/2868 | 8.69 | 1.11(0.93,1.31) | 1.04(0.88,1.24) | 1.03(0.85,1.24) |
| G2*(26.40-39.56mg/dl) | 273/3054 | 8.79 | 1.11(0.94,1.32) | 1.07(0.94,1.32) | 1.06(0.89,1.26) |
| G1*(<26.40mg/dl) | 265/3156 | 8.29 | 1.00 (Ref.) | 1.00 (Ref.) | 1.00 (Ref.) |
| G1(<26.40mg/dl) | 50/410 | 12.74 | 1.40(1.04,1.90) | 1.34(0.97,1.84) | 1.34(0.97,1.84) |
| G2(26.40-39.56mg/dl) | 75/538 | 14.73 | 1.74(1.34,2.24) | 1.57(1.19,2.08) | 1.56(1.18,2.07) |
| G3(39.57-54.65mg/dl) | 90/581 | 16.49 | 2.04(1.61,2.59) | 1.80(1.38,2.35) | 1.78(1.35,2.34) |
| G4(≥54.66mg/dl) | 129/806 | 16.77 | 2.10(1.70,2.59) | 1.91(1.50,2.43) | 1.86(1.41,2.45) |
| *P*-trend |  |  | <0.0001 | <0.0001 | <0.0001 |

G1*-G4*were participants without diabetes；G1-G4 were participants with diabetes.

Model 1: adjusted for age and sex.

Model 2: included variables in model 1 and further SBP, LDL-C, HDL-C, BMI, hs-CRP, eGFR, family history of CVD, smoking status, alcohol consumption, physical exercise habits, the use of antihypertensive drugs, the use of glucose-lowering drugs, and the use of lipid-lowering drugs.

Model 3: included variables in model 2 and further the RC at baseline.

**Table S5** Comparison of predictive ability for CVD between non-HDL-C, RC_2006_, RC_2010_ and Cum-RC

|  | **C-index** | **NRI** |  | **IDI, (%)** |  |
| --- | --- | --- | --- | --- | --- |
|  | **Estimate**  **(95% CI)** | **Estimate**  **(95% CI), %** | **P-value** | **Estimate**  **(95% CI), %** | **P-value** |
| Base model* | 0.6524(0.6386, 0.6662) | Ref. |  | Ref. |  |
| Base model + non-HDL-C | 0.6546(0.6388, 0.6664) | 8.03(2.49, 13.56) | 0.0046 | 0.017(0.01, 0.03) | 0.0584 |
| Base model + RC_2006_ | 0.6573(0.6437, 0.6710) | 9.42(3.89, 14.95) | 0.0009 | 0.036(0.01, 0.06) | 0.0121 |
| Base model + RC_2010_ | 0.6554(0.6417, 0.6690) | 8.85(3.33, 14.38) | 0.0018 | 0.030(0.01, 0.06) | 0.0657 |
| Base model + Cum-RC | 0.7028(0.6837, 0.7256) | 11.83(6.57, 17.09) | <0.0001 | 0.044(0.01, 0.08) | 0.0083 |

NRI, net reclassification index. IDI, integrated discrimination improvement.

*Base model was adjusted for age, sex, smoking status, blood pressure, diabetes, waist circumference, high-density lipoprotein cholesterol, family history of CVD, and the use of antihypertensive drugs.
